# Changes in sociocultural stressors, protective factors, and mental health for US Latina mothers in a shifting political climate

**DOI:** 10.1371/journal.pone.0273548

**Published:** 2022-08-25

**Authors:** Amy L. Non, Elizabeth S. Clausing, Kimberly L. D’Anna Hernandez

**Affiliations:** 1 Department of Anthropology, University of California, San Diego, La Jolla, CA, United States of America; 2 School of Global Integrative Studies, University of Nebraska, Lincoln, Lincoln, NE, United States of America; 3 Department of Psychology, Marquette University, Milwaukee, WI, United States of America; Shimonoseki City University, JAPAN

## Abstract

**Background:**

To investigate changes in sociocultural stressors and protective factors, and mental health in Latina mothers before and after the 2016 Republican presidential nomination.

**Methods:**

We examined changes in sociocultural stressors, protective factors, and mental health from two prospective cohorts of Latina mothers from interior and border US cities (Nashville, TN, *n* = 39 and San Diego, CA, *n*s range = 78–83; 2013–2020).

**Results:**

We identified significant longitudinal increases in depression, anxiety, and perceived stress in the border city, and reductions in protective factors (e.g., optimism, social support, and familism) across sites. Discrimination varied by location, and was associated with higher stress only at baseline in the border city, and with higher anxiety in the interior city at follow-up. Acculturative stress was consistently associated with worse mental health across time points in the border city. Various protective factors were associated with reduced stress and anxiety across time points in both cities.

**Discussion:**

We identified decreased mental health at the border city, and reduced protective factors in Latina mothers across both study sites in the years following the 2016 presidential nomination, during a time of shifting sociopolitical climate. We also identify increased acculturative stress and discrimination over time, particularly at the border city. Interventions to maintain and enhance psychosocial protective factors amongst Latina mothers are warranted.

## Introduction

The well-being of Latinx Americans in the US is especially vulnerable in the current political climate. Historically, Latinx people have been disadvantaged on numerous fronts, including access to quality education, job security, and healthcare [1], while simultaneously being confronted with racism [2]. In recent years, Latinx people face heightened stereotypes, bigotry, and fears resulting from increasingly racist language and anti-immigrant sentiments in politics and the media [3]. Latinx immigrants additionally experience inhumane detention and deportation practices and immigration policies including executive orders and policies that deny asylum to refugees and separate parents from their children [4]. Even the perception or threat of major policy changes can harm immigrant mental health [5]. Hate crimes have also increased, potentially motivated by these attitudes and policies [4, 6], making the US an increasingly dangerous place for Latinx Americans to live.

This sociopolitical climate may be particularly toxic for the mental health of Latina mothers who struggle with discrimination and/or fears about deportation for themselves or their family members. In fact, Latina mothers nationwide experienced increases in preterm birth rates above expected levels after 2016 [7], which may have been related to increased sociopolitical stress and anxiety in the new policy environment of the Trump presidency. These mothers are also likely facing heightened racial discrimination, which has been significantly associated with worse mental health across studies of racial/ethnic minorities [8]. Even before the Trump-era policy changes, perinatal Latina mothers reported 2–3 times the rates of depression and anxiety compared to women in the general U.S. population [9].

**Fig 1:**
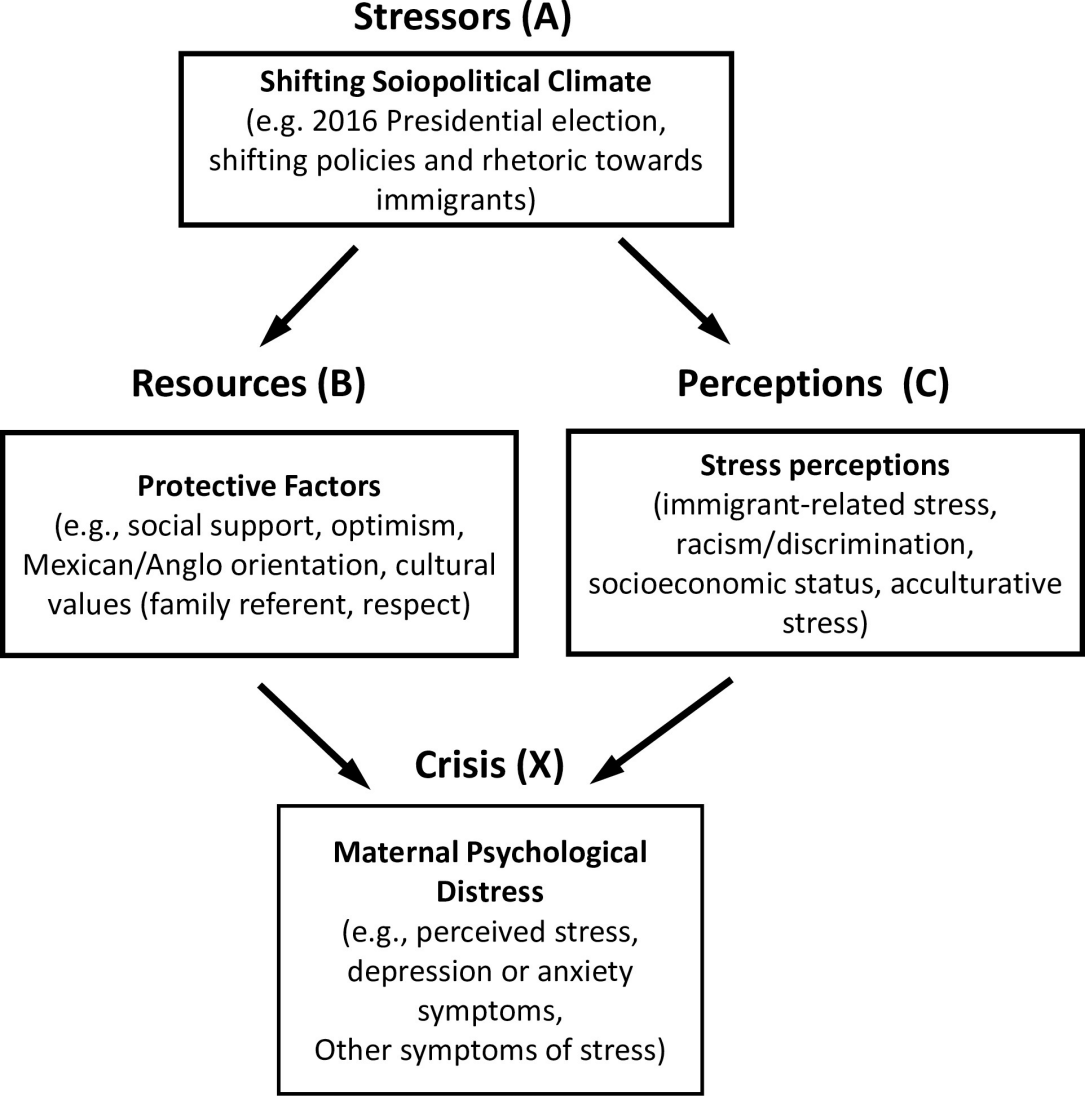
ABC-X family stress model. This conceptual model shows how our measures fit with the ABC-X family stress model. A shifting sociopolitical climate hostile towards Latina families can influence both perceptions of stress and resources to cope with stressors. Both resources and perceptions in turn influence maternal psychological responses. If protective factors are too low, and stress perceptions are too high, this can lead to a crisis of maternal distress, which ultimately impacts the whole family.

We investigate the stressors faced by Latina mothers in this study within a family stress theory framework, first conceptualized by Reuben Hill’s ABC-X model [10]. This model demonstrates how families are influenced not just by the stressful events alone (A), but also by the family’s resources to cope with it (B), and the family’s appraisal of the event (C). Thus, the stress or crisis event, X, is ultimately a function of these other factors (Fig 1). Here, we recognize that in addition to high levels of stress exposures, Latina mothers historically have been buffered by high levels of protective factors, such as close ties to family (familism) and other Latinx cultural values. Familism and social support can buffer against depressive symptoms and overall promote psychological well-being [11]. Optimism also buffers hopelessness, and is likely higher among immigrants who typically emigrate in search of a better future for their families [12]. However, it is unclear for Latina mothers if these protective factors erode with time lived in the US, or if they continue to protect against mental health problems, though some work has suggested that values most strongly rooted in cultural identity are fairly stable over time, particularly in adulthood and for women, and may even strengthen after giving birth [9, 13, 14].

It is not yet clear if Latina mothers report increasing discrimination or stress nationally. Implementation of immigration policies and racist attitudes are not uniform throughout the country, and experiences may vary particularly between traditional immigrant destinations closer to the border versus newer immigrant destinations in the interior of the country. Not all Americans may be reacting in similar ways; the political climate, as amplified by the prior presidential administration, may have simultaneously galvanized racists and mobilized anti-racists in support of immigrants [15], perhaps even fostering a climate of defiance and solidarity among some immigrant communities [16]. Thus, we investigate the mental health effects of this shifting sociopolitical climate in Latina mothers in two distinct samples in border and interior regions of the US (San Diego, CA and Nashville, TN). We hypothesized that in both locations, following a shift in sociopolitical climate after President Trump’s selection as the Republican candidate for president: 1) discrimination and immigrant-related stressors would increase, 2) protective factors would decrease, 3) mental health would decline, and 4) immigrant-related stressors and protective factors would associate with mental health differently before and after Trump’s candidacy (possibly due to shifting levels of these factors over time). In order to address these hypotheses, a longitudinal analysis was conducted in both samples evaluating the same mothers before and after Trump’s candidacy.

## Methods

### Study populations

**Fig 2:**
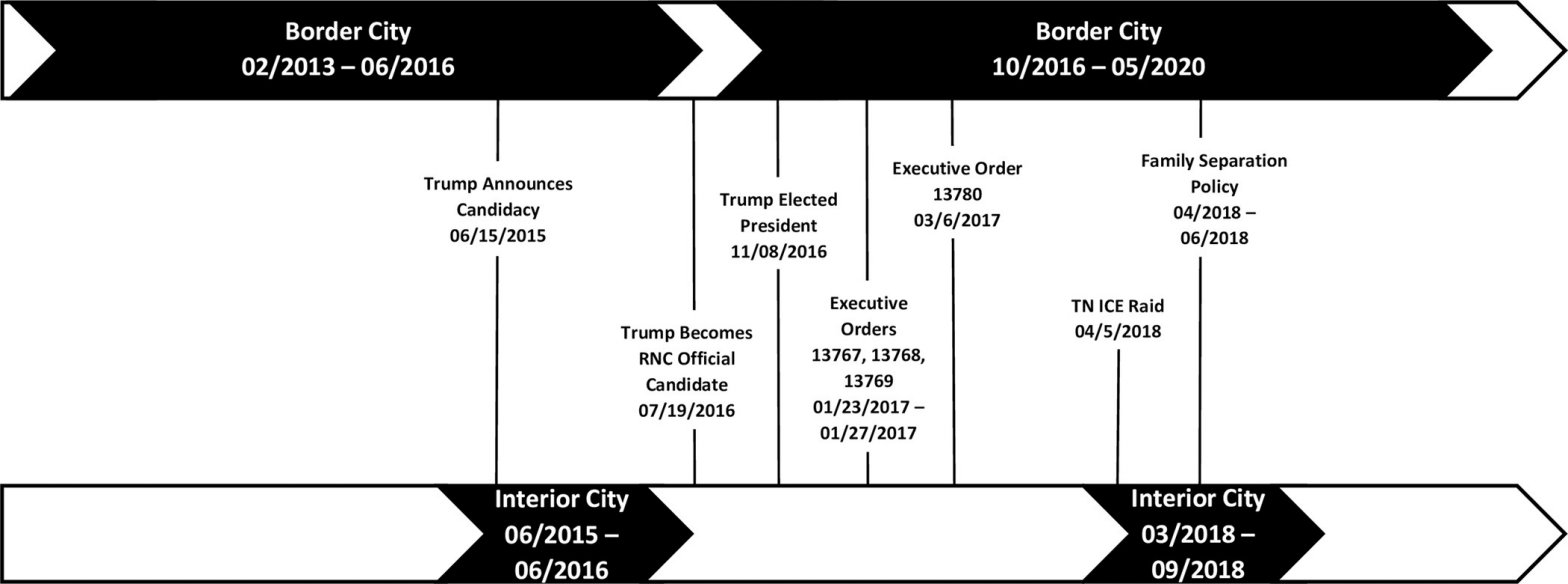
Timeline of study sample collection. These two timelines show the timing of sample collection in the two locations of our study in relation to federal policies shared by both sites (shown by lines connecting to both timelines) and a local event (in the interior city) that impacted immigrants during the post-candidacy period. Three Executive Orders (13767, 13768, and 13769) were issued by the President within the last week of January 2017. These orders collectively included demands for a border wall (13767), expanded use of detention of immigrants (13768), limited access to asylum, enhanced enforcement along the border and increased the number of ICE agents (13768, 13769), prohibited sanctuary jurisdictions from receiving federal funding (13768), and limited travel from Muslim majority countries (13769). Additionally, a policy specifically aimed at separating families at the border was enacted in spring of 2018. In TN specifically, an ICE raid on a meatpacking plant occurred a few hours away, possibly the largest raid of Trump administration with 86 individuals detained and 11 arrested. All together, these policies sent a collective message that immigrants were not welcome and increased anti-immigrant media attention during this time.

Our samples are drawn from two longitudinal studies of Latina mothers and children in the US in two geographic regions. Both studies collected samples before and after the announcement of Trump as the official Republican candidate for president on July 19^th^, 2016 (hereafter referred to as baseline and follow-up). The nomination date was chosen because this marked a shift in media and public attitude towards Trump as a serious potential president. The timing of both studies in relation to immigrant-related events can be found in Fig 2.

The first sample is drawn from a birth cohort near the US-Mexico border in Southern California [17], hereafter referred to as the “border city.” Pregnant mothers were recruited from a community clinic, between 2013 and 2019. Participants were of Mexican descent, 18 years or older, less than 15 weeks pregnant, no current illicit substance or tobacco use, and singleton pregnancy. Surveys were conducted in participant’s preferred language (67% English and 33% Spanish). Informed and written consent were provided by all participants and all procedures were approved by the Institutional Review Board at (California State University San Marcos).

For all analyses in the border city (*n* = 83), the sample was restricted to those who participated in the 6-week postpartum assessment at baseline (February 2013-July 2016) and again in the 3-year follow-up (Dec 2016-May 2019).

The second dataset draws from a study focused on stress in Latina immigrant mothers and their children located in Nashville, TN [18]. This study is referred to as “interior city.” Data were collected at baseline and follow-up (June 2015-June 2016, *n* = 82; March-September 2018, *n* = 39) from the same individuals. Longitudinal analyses include 39 who participated at both time points. The baseline subsample (*n* = 39) did not differ significantly from the baseline full sample (*n* = 82) on any measured demographics, including maternal age, education, years in US, legal status, parity, marital status, country of birth (mother or child), or children’s gender or age. Participants were recruited from community centers with subsequent snowball sampling. Inclusion criteria were self-described Latina, foreign-born immigrant mothers above age 18, with a child between the ages of 6–13. Data were collected via structured individual interviews all conducted in Spanish, with questions derived from preliminary focus groups [19]. Informed oral consent was provided by all participants, and Vanderbilt University Institutional Review Board approved all protocols.

### Population demographics

Participants self-reported all demographic characteristics, including maternal age, education, country of birth, years in US, subjective SES, legal status, parity, marital status, and children’s gender, age, and country of birth (Table 1).

**Table 1:** Demographics of study samples at baseline and follow-up in interior and border cities.

	Longitudinal Comparisons			
	Interior City		Border City	
Demographics	Baseline (*n* = 39)	Follow-up (*n* = 39)	Baseline (*n* = 83)	Follow-up (*n* = 83)
Time between sample collections (months), mean±sd (range)	--	31.3±3.2		34.4±5.0
		(20.0–37.0)		(34.0–61.0)
**Maternal Characteristics**				
Age in years, mean±sd (range)	34.7±4.8	37.4±4.8	28.2±5.0	32.7±7.2
	(26.0–43.0)	(29.0–47.0)*	(18.0–39.0)	(22.0–43.0)*
Education, *n* (%)				
< HS Degree	24 (61.5%)	24 (61.5%)	35 (42.2%)	35 (42.2%)
Mother’s country of birth, *n*				
(%)				
Mexico	36 (92.3%)	36 (92.3%)	61 (73.5%)	61 (73.5%)
Other Central/South	3 (7.7%)	3 (7.7%)	--	--
America				
United States	--	--	22 (26.5%)	22 (26.5%)
Years in US, mean±sd (range)	12.5±3.4	15.0±3.4	16.0±7.0	20.0±7.2
	(4.0–20.0)	(6.7–22.4)*	(0.5–31.0)	(8.0–35.0)*
Mother’s Subjective SES,	3.80±2.1	4.4±1.6	--	--
mean±sd (range)	(1.0–9.0)	(1.0–8.0)	--	--
Legal status, *n* (%)				
Undocumented	34 (87.2%)	33 (84.6%)	--	--
Documented	5 (12.8%)	5 (12.8%)	--	--
Unknown	0 (0%)	1 (2.6%)	--	--
Undocumented Partner, *n* (%)	17 (47.2%)	12 (34.3%)	--	--
Parity, *n* (%)				
0	0 (0%)	0 (0%)	16 (19.3%)	--
1	0 (0%)	0 (0%)	24 (28.9%)	--
2	9 (23.1%)	9 (23.1%)	24 (28.9%)	--
3	20 (51.3%)	15 (38.5%)	13 (15.7%)	--
4 or more	10 (25.6%)	11 (28.2%)	6 (7.2%)	--
Unknown	0 (0%)	4 (10.0%)	0 (0%)	--
Marital Status, *n* (%)				
Married/Living Together	36 (92.3%)	30 (76.9%)	66 (79.3%)	65 (78.3%)
Single (Never Married)	2 (5.1%)	4 (10.3%)	11 (13.8%)	4 (4.8%)
Widowed	1 (2.6%)	1 (2.6%)	0 (0%)	0 (0%)
Divorced/Separated	0 (0%)	1 (2.6%)	6 (6.9%)	8 (9.7%)
Unknown	0 (0%)	3 (7.7%)	0 (0%)	6 (7.2%)
**Child Characteristics**				
Child’s Gender (female), *n* (%)	22 (56.4%)	22 (56.4%)	28 (48.3%)	28 (48.3%)
Child’s age, mean±sd (range)	8.28±1.99	10.70±1.94	6.20±1.85	3.75±0.43
	(5.0–13.0)	(8.0–15.0)	(3.3–13.0)	years
	years	years	weeks	(2.9–5.2)
Child’s country of birth, *n* (%)				
United States	28 (73.7%)	28 (73.7%)	58 (100%)	58 (100%)
Mexico/Other	10 (26.3%)	10 (26.3%)	--	--

*Indicates significant difference in means or frequencies baseline- vs. follow-up (*p*<0.05). In the interior city, some frequencies add to 38 or 39, depending on missing data.

### Study settings

The border city study was located in San Diego, CA. Due to its proximity to Mexico (within 50 miles), it is a traditional immigrant border destination with a large Mexican population (up to 46% in some regions) and a long history of community support and integration. However, due to its border location, immigrants also face increased presence of border patrols, with San Diego historically as one of the busiest border patrol sectors in the nation [20]. Californian Latinos are also unique in showing a higher level of political knowledge than other states, which could make these participants more aware of harmful political rhetoric and new policies directed towards immigrant groups [21].

The interior city study context is located in Nashville, TN, a city with a growing Latinx immigrant population, currently 10.5% [22]. Tennessee was the state with the largest percent growth (143%) of children living with immigrant parents from 2000–2010; however, the growth has since slowed, as immigration has slowed nationally [23]. Nashville has experienced rapidly changing policies towards immigrants leading to much uncertainty and anxiety about their status and safety, including a highly publicized ICE raid a few hours away, just a week after our follow-up sample collection began (see Fig 2).

Comparison across these two study sites presents an opportunity to explore stress experiences in two distinct US contexts–a traditional immigrant border destination and newer interior immigrant destination. The dynamics and timing of immigration and enforcement in these regions has been very different historically, and thus comparison across these cities provides an opportunity to explore unique Latina immigrant experiences across the nation. While the scales and surveys used across both cities differed, they represent similar concepts across study sites (e.g., discrimination and immigrant-related stressors, protective factors, and mental health outcomes).

### Sociocultural stressors

#### Interior city

A 10-item measure of *immigrant-related stress* was used, with questions about emigrating plus immigrant stressors in the past year [19]. An example question from the *immigrant-related stress* scale is “Answer yes/no: When I emigrated to the US, I felt stressed because I separated from spouse or children.” A 7-item scale assessed personal and vicarious *discrimination stress* experiences. An example of the *discrimination stress scale* is “How often did you feel that you experienced discrimination in your neighborhood because you are an immigrant?” Items in both sociocultural stress scales are listed in S1 Table and described elsewhere [19]. Women also self-reported about frequency of arrests and harassment by police. We also asked two open-ended questions about how women felt when seeing “Make American Great Again Hats” in the wake of the 2016 election, and any behavior changes they may have implemented as a result of the Trump election.

#### Border city

The Social, Attitudinal, Familial, and Environmental Stress Scale (SAFE) is a 24-item scale that assesses *acculturative stress* [24]. Two example questions are, “It bothers me that family members I am close to do not understand my new values” and “Many people have stereotypes about my culture or ethnic group and treat me as if they are true.” Responses ranged from 1 (not stressful) to 5 (extremely stressful).

The *Discrimination Stress Scale* (DSS) is a 14-item scale that assesses how often racial/ethnic discrimination occurs in everyday life [25]. An example question is, “How often do others lack respect for you because of your race or ethnicity?” Responses ranged from 1 (never) to 4 (very often).

### Protective factors

#### Interior city

The revised 10-item Life Orientation Test (LOT-R) measured *generalized optimism* [26]. An example question of the LOT-R is “In uncertain time, I usually expect the best.” Responses included 5 options, ranging from “I agree a lot” to “I disagree a lot,” with items 3, 7, 9 reverse scored, and 2,5,6,8 removed as fillers. An 11-item scale measured *social support and connection*. Six items relate to emotional and tangible support and five items relate to social connection from friends and family, adapted from Berkman and Syme Social Network Index [27]. An example question of the *social support and connection scale* is “I have friends or family with whom I can talk about my feelings or problems.”

#### Border city

The *Acculturation* Rating Scale for Mexican-Americans Revised is a 30-item questionnaire with two subscales, Mexican (MOS) and Anglo (AOS) Orientation [28]. An example question from the MOS is “My friends, while I was growing up, were of Mexican origin.” An example question from the AOS is “My thinking is done in the English language.” Responses ranged from 1 (not at all) to 5 (extremely often of almost always).

The Mexican American *Cultural Values Scale* (MACVS) [29] has 6 enculturative value scales (familism support, obligation, referent and respect, religion, traditional gender roles), 3 acculturative value scales (material success, independence and self-reliance, and competition), and subscales of familism and enculturative values. Examples of enculturative questions from the 6 subscales listed above respectively are, “Parents should teach their children that the family always comes first,” “If a relative is having a hard time financially, one should help them out if possible,” “When it comes to important decisions, the family should ask for advice from close relatives,” “Children should respect adult relatives as if they were parents,” “One’s belief in God gives inner strength and meaning to life,” and “A wife should always support her husband’s decisions, even if she does not agree with him.” Examples of acculturative questions from the 3 subscales above respectively are, “Money is the key to happiness,” “When there are problems in life, a person can only count on him or herself,” and “Personal achievements are the most important things in life.” Participants responded how much they believed each statement from 1 (not at all) to 5 (completely).

### Mental health measures

#### Interior city

A validated Spanish version of the Hospital Anxiety and Depression Scale (HADS) was used only at follow-up [30]. Self-reported symptoms of stress were also measured at both time points (see S1 Table). HADS scores were classified as “normal” (0–7), “borderline case” (8–10) or “case” (11–21) for both *anxiety and depression*. The *Perceived Stress* Scale (PSS) [31] was administered to mothers in the interior city only at follow-up, and classified as low (< = 13) moderate (14–26), and high stress (27–40).

#### Border city

For *perceived stress*, the PSS was used [31]. Scores were classified in the same way as for the interior city. An example item is, “In the last month, how often have you felt that you were unable to control the important things in your life?” Participants responded how much they agreed with each statement from 1 (very strongly disagree) to 5 (very strongly agree).

The Center for Epidemiologic Studies Depression Scale (CES-D) assessed *depressive symptoms* in the last week [32]. Scores were classified as low (< = 9), moderate (10–15), or high (> = 16). An example item is, “I felt I could not shake off the blues even with help from my family or friends. Responses ranged from 0 (rarely or none of the time) to 3 (most or all of the time).

The State Trait Anxiety Inventory (STAI–Form Y-1) measured participants’ *state anxiety* [33]. STAI scores were classified as none or low (20–37), moderate (38–44), and high (45–80). An example item is, “I am presently worrying over possible misfortune.” Participants responded on frequency of feelings from 1 (never) to 4 (very much so).

Reliability scores and questions in all scales used in the interior city are available in S1 Table. All scales used in the border city show good reliability scores with high α’s (ranging 0.82–0.96) and have been used with pregnant women of Mexican descent [17, 34–37]. The scales used across both cities differed, but addressed parallel concepts such as immigrant-related stress and mental health. However, the protective factors measured in each city were more distinct, whereas the border city used culturally relevant measures of acculturation and cultural values, and the interior city used more generalizable measures of optimism and social support.

### Statistical analysis

#### Longitudinal analyses in border and interior cities

Paired sample t-tests investigated differences in means of continuous measures in the same mothers at baseline and follow-up. McNemar tests for paired samples were conducted on all categorical variables. All data analyzed in this study will be made available upon request.

#### Regression analyses of mental health at baseline and follow-up

Multiple linear regressions were conducted to determine associations between sociocultural stressors or protective factors with continuous mental health outcomes cross-sectionally at both baseline and follow-up time points in both cities. In the interior city, additional logistic regressions for presence of symptoms of stress were conducted at both time points, but anxiety, depression, and perceived stress were only collected and analyzed with linear regressions at the follow-up time point. All regressions were adjusted for maternal age and years in the US.

## Results

### Sample demographics

See Table 1 for sample demographics. In brief, the interior city sample at baseline had a mean age of 34 years for mother and 8.3 years for children, with the majority of mothers having less than a high school degree (61.5%), mostly married (92.3%), primarily born in Mexico (92.3%), lived in the US for an average of 12.5 (range 4–20 years), and the vast majority (87.2%) were undocumented. In the border city at baseline, the mean age for mothers was 30 years, and for children was 6.2 years, 42.2% of mothers had less than a high school degree, 79.3% were married, 73.5% were born in Mexico and 26.5% were US-born, on average they all lived in the US 16.0 years (range 0.5–31), and data on documentation status was not collected.

### Longitudinal analyses

#### Sociocultural stressors

*Interior city*. There were small but significant decreases from baseline to follow-up in levels of immigrant-related stress and overall discrimination stress (*p* = 0.04). However, reported frequency of women being arrested or harassed by police both increased over time (Table 2).

**Table 2:** Changes in sociocultural stressors, protective factors, and mental health measures in the interior city from baseline to follow-up.

	Baseline	Follow-up	Test Statistic
	*n* = 39	*n* = 39	(*p*-value)^‡^
**Sociocultural Stressors**			
Immigrant-related stress score, mean±sd (range)	0.47±0.23 (0.1–0.9)	0.41±0.2 (0–0.7)	**-2.12 (0.04)** ^*^
Police Arrests, No., %	0, 0%	3, 7.7%	1.33 (0.25)
Police Harassment, No., %	1, 2.6%	6, 15.4%	2.29 (0.13)
Overall Discrimination Score, mean±sd, (range)	0.44±0.27 (0–1.0)	0.36±0.24 (0–0.9)	**-2.14 (0.04)** ^*^
**Protective Factors**			
Optimism, mean±sd (range)	17.70±3.00 (12.0–24.0)	13.60±2.28 (8.0–18.0)	**-6.62 (<0.01)** ^**^
Social Support/Connection mean±sd (range)	2.91±0.34	2.20±0.55	1.79 (0.08)
	(2.2–3.7)	(1.0–3.4)	
**Mental Health Measures**			
Anxiety (HADS), mean ± sd, (range)	--	6.08±3.58 (0.0–14.0)	--
Depression, (HADS), mean±SD, (range)	--	6.55±3.24 (1.0–16.0)	--
Perceived Stress, mean±sd (range)	--	17.90±4.30 (8.0–26.0)	--
Symptoms of Stress			
Tiredness, *n* (%)	25 (64.1%)	34 (87.1%)	**4.27 (0.04)** ^*^
Sickness, *n* (%)	17 (43.6%)	22 (56.4%)	0.76 (0.38)
Aging too quickly, *n* (%)	15 (38.5%)	22 (56.4%)	1.57 (0.21)
Energy Level, *n* (%)	17 (43.6%)	23 (59.0%)	1.14 (0.29)
Staying Asleep, *n* (%)	19 (48.7%)	18 (46.2%)	0.00 (1.00)
Falling Asleep, *n* (%)	21 (53.8%)	18 (46.2%)	0.17 (0.67)
Fear, *n* (%)	16 (41.0%)	13 (33.3%)	0.24 (0.63)
Anxiety/Depression, *n* (%)	24 (61.5%)	13 (33.3%)	0.41 (0.52)
Anger/Frustration, *n* (%)	26 (66.7%)	21 (53.8%)	0.84 (0.36)

^‡^Test statistic is a paired *t*-test statistic for baseline vs. follow-up comparison of continuous variables, and McNemar chi squared for test of categorical paired data.

*Indicates significant difference in means or frequencies between baseline vs. follow-up at *p* < 0.05 and

**p < 0.01.

*Border city*. Reports of perceived discrimination and acculturative stress both increased significantly from baseline to follow-up (*p* = 0.005 and *p* = 0.002, respectively), but there were no changes in acculturation (Table 3).

**Table 3:** Changes in sociocultural stressors, protective factors, and mental health measures for the border city from baseline to follow-up (*n*s range 78–83).

	Longitudinal Analyses		
	Baseline	Follow-up	Test Statistic
			(*p*-value)^‡^
**Sociocultural Stressors mean±sd (range)**			
Discrimination	1.28±0.32 (1.00–2.21)	1.40±0.40 (1.00–2.42)	**2.899 (0.005)** ^**^
Acculturative Stress	19.96±14.38 (0.00–58.00)	25.03±13.54 (0.00–65.00)	**3.179 (0.002)** ^**^
**Protective Factors, mean±sd (range)**			
Mexican Orientation	4.24±0.55 (0.02–5.00)	4.24±0.53 (2.00–5.00)	-0.051 (0.959)
Anglo Orientation	2.75±1.06 (1.23–5.00)	2.82±1.02 (1.31–4.77)	1.254 (0.213)
*Overall Enculturative Values (Mexican)*	3.82± 0.57	3.64± 0.53	**-3.675 (<0.001)** ^***^
Overall Familism	4.43±0.47 (3.17–5.00)	4.33±0.54 (3.17–5.00)	**-2.917 (0.005)** ^***^
Family support	4.43±0.47 (2.00–5.00)	4.33±0.54 (2.40–5.00)	-1.476 (0.144)
Family obligation	3.90± 0.68 (2.00–5.00)	3.62±0.71 (2.00–5.00)	**-3.298 (0.001)** ^**^
Family referent	3.64±0.81 (2.30–5.00)	3.42±0.76 (2.30–5.00)	**-2.245 (0.028)** ^*^
Respect	4.14±0.62 (2.30–5.00)	3.98±0.59 (1.70–5.00)	**-2.518 (0.014)** ^*^
Religion	4.21±0.69 (1.20–5.00)	4.16±0.75 (1.00–4.60)	-0.794 (0.430)
Gender Roles	2.69±0.82 (2.40–5.00)	2.30±0.61 (2.75–5.00)	**-3.990 (<0.001)** ^***^
*Overall Acculturative Values (Anglo)*	2.61±0.59 (1.20–4.20)	2.60±0.51 (1.50–4.00)	-0.064 (0.949)
Material Success	1.47±0.57 (1.00–2.60)	1.57±0.63 (1.00–3.20)	1.43 (0.157)
Independence and self-reliance	3.44±0.77 (1.40–5.00)	3.48±0.62 (1.00–5.00)	0.44 (0.658)
Competition and achievement	2.84±0.91 (1.00–5.00)	2.70± 0.82 (1.30–4.50)	-1.41 (0.162)
**Mental Health measures mean±sd (range)**			
Anxiety	30.96±8.95 (20.00–58.00)	34.06±11.10 (20.00–70.00)	**2.190 (0.032)** ^*^
Depression	8.92±7.23 (0.00–31.00)	10.44±8.08 (0.00–32.00)	1.592 (0.111)
Perceived Stress	17.80±7.61 (2.00–38.00)	21.92±7.65 (2.00–41.00)	**4.810 (<0.001)** ^***^

‡Test statistic is a paired *t*-test statistic. Bold font indicates significant change from baseline to follow-up at

*p≤0.05

**p<0.01, and

***p<0.001.

#### Protective factors

*Interior city*. In the follow-up, women reported significantly decreased optimism (*p*<0.01) and marginally decreased social connection/support (*p* = 0.08) (Table 2).

*Border city*. Overall adherence to enculturative values decreased significantly at follow-up, including respect, gender roles, overall familism, and two of its subscales (all *ps*<0.04) (Table 3). Adherence to acculturative (US mainstream) values did not change.

#### Mental health

*Interior city*. At follow-up, mothers reported significantly higher levels of tiredness relative to baseline (64% vs 87%, McNemar $X^{2}$ = 4.27, p = 0.04), but no difference in other symptoms of stress (Table 2). When mental health measures were classified into diagnostic groups, a high proportion reported borderline or clinically diagnostic levels of depressive symptoms (29%), anxiety symptoms (39.5%), and moderate levels of perceived stress (86.8%) at follow-up. No women reported high stress.

When mothers in the interior city were asked how their behaviors changed since Trump became president, or how they felt when seeing “Make America Great Again” hats, they reported overwhelming sentiments of feeling sad, unsafe, or threatened. Behavior changes were mostly described as fear-based; e.g., being more cautious when going out, limiting their outings, etc. For example, one mother said, “Going out is no longer the same. I go out of necessity. Otherwise, I’d be home.” Other responses include fear or avoidance of large crowded events, of being out too late to avoid being seen by the police, following traffic laws strictly (e.g., respecting speed limits), and generally avoiding anything that could jeopardize their residence in the US. These sentiments corresponded with the high levels of depressive and anxiety symptoms and perceived stress at follow-up.

*Border city*. Levels of anxiety and perceived stress (but not depression) significantly increased at follow-up (anxiety: *t*(77) = 2.190, CI: 0.26, 5.47, perceived stress: *t*(77) = 4.810, CI:2.64, 6.38; Table 3). When classified into diagnostic groups, higher levels at follow-up vs. baseline were found in moderate/high depressive symptoms (40.0%) vs. 36.5%), anxiety (34.0% vs 20.8%), and perceived stress (84.4% vs 61.2%).

#### Role of sociocultural stressors and protective factors on mental health at baseline and follow-up

*Interior city*. Discrimination stress associated with marginally higher levels of anxiety at follow-up. Social support/social connection was significantly associated with lower levels of perceived stress, while optimism was significantly associated with lower levels of depression at follow-up (Table 4).

**Table 4:** Linear regression models of maternal sociocultural variables explaining mental health symptoms at follow-up in the interior city (n = 34).

		*Mental Health Outcomes*					
Models		HADS Anxiety		HADS Depression		PSS	
Maternal variables		Adj *R*^2^	*B*(SE)	Adj *R*^2^	*B*(SE)	Adj *R*^2^	*B*(SE)
**Sociocultural Stressors:**							
	Immigrant-related stress	-0.023	2.96 (3.48)	-0.044	1.67 (3.09)	-0.024	4.65 (4.18)
	Discrimination stress	*0*.*059*	*4*.*70 (2*.*47)*	0.016	3.37 (2.22)	-0.009	4.11 (3.19)
**Protective Factors:**							
	Social support/ connection	0.681	-0.48 (1.16)	-0.047	-0.43 (1.02)	**0.061**	**-2.74 (1.31)**
	Optimism	-0.002	-0.33 (0.27)	**0.094**	**-0.53 (0.23)**	0.008	-0.51 (0.33)

The models are adjusted for maternal age and years in the US. **Bolded** values indicate *p*<0.05. *Italicized* values indicate *p*<0.10.

Among the symptoms of stress analyzed at baseline, we detected significant associations between immigrant-related stress and symptoms of sickness, aging too quickly, and trouble staying asleep, but only the association with staying asleep remained marginally significant at follow-up (S2 Table). Discrimination stress associated with increased sickness and anger/frustration at baseline, and was marginally associated with decreased tiredness, and increased fear at follow-up. Among protective factors, social support/connection was significantly associated with decreases across five of the symptoms at baseline only (S2 Table).

*Border city*. Acculturative stress significantly predicted more symptoms of depression, anxiety, and perceived stress at baseline, but only more depressive symptoms and perceived stress at follow-up (Table 5). At baseline, increased Mexican Orientation was associated with less perceived stress, but not with anxiety or depression, while discrimination was associated with more stress (Table 5). However, neither were related to any mental health measures at follow-up. Of the cultural values, neither overall enculturative values nor familism was associated with maternal mental health at either timepoint. However, adherence to the traditional values of respect and family reference were associated with less anxiety at follow-up, but not baseline (Table 5). In contrast, adherence to the traditional value of gender roles was only associated with more perceived stress at baseline, but not follow up (Table 5).

**Table 5:** Multiple regression models of maternal sociocultural stressors and protective factors explaining mental health variables at baseline and follow-up in border city (*n* = 78–84 both baseline and follow-up).

Models						
Mental Health Measures		Depression symptoms		Anxiety symptoms		Perceived Stress
	Adj *R*^2^	*B*(SE)	Adj *R*^2^	*B*(SE)	Adj *R*^2^	*B*(SE)
		CI		CI		CI
**Acculturative Stress** Baseline						
	**0.166**	**0.190 (0.047)**	**0.136**	**0.204 (0.063)**	**0.167**	**0.194 (0.051)**
		0.097–0.283***		0.078–0.329**		0.093–0.296***
Follow-up	**0.087**	**0.157 (0.059)**	0.018	0.110 (0.088)	**0.091**	**0.156 (0.058)**
		0.039–0.274**		-0.064–0.285		0.041–0.270**
**Discrimination** Baseline						
	0.061	5.036 (2.211)	0.081	4.025 (2.986)	**0.127**	**5.301 (2.329)**
		0.633–9.439		-1.923–9.973		0.663–9.940*
Follow-up	0.026	1.972 (2.056)	0.012	2.985 (2.986)	0.026	1.456 (2.009)
		-2.112–6.057		-2.944–8.914		-2.461–5.5463
**Mexican Orientation** Baseline						
	0.072	-1.822 (1.325)	0.092	-0.819 (1.741)	**0.116**	**-3.139 (1.400)**
		-4.458–0.813		-4.280–2.643		-5.992- -0.355*
Follow-up	0.017	0.477 (1.644)	0.009	2.022 (2.387)	0.023	1.131 (1.597)
		-2.790–3.743		-2.718–6.762		-2.040–04.303
**Family referent**						
Baseline	0.047	0.694 (0.934)	0.057	-0.614 (1.225)	0.039	0629 (1.001)
		-1.164–2.553		-2.602–2.275		-1.363–2.621
Follow-up	0.017	-0.309 (1.102)	0.041	*-3*.*030 (1*.*560)*	0.023	-0.902 (1.066)
		-2.499–1.882		*-6*.*129–0*.*069ǂ*		-3.021–1.216
**Respect**						
Baseline	0.055	1.317 (1.194)	0.057	0.129 (1.573)	0.034	0.134 (1.301)
		-1.060–3.693		-3.002–3.261		-2.456–2.724
Follow-up	0.030	-1.636 (1.453)	0.057	**-4.785 (2.067)**	0.044	-2.309 (1.398)
		-2.742–3.192		-8.891- -0.679*		-5.087–0.469
**Gender Roles**						
Baseline	0.066	1.263 (0.875)	0.069	1.164 (1.152)	0.089	**2.035 (0.929)**
		-0.479–3.006		-1.129–3.458		0.186–3.884*
Follow-up	0.017	0.354 (1.052)	0.002	-0.072 (1.523)	0.017	-0.452 (1.017)
		-1.736–2.443		-3.097–2.953		-2.471–1.568

The models are adjusted for maternal age and years in the US.

^ǂ^p<0.06

**p<0.01

***p<0.001. Adj = adjusted.

## Results summary

Overall, both cities showed a similar decrease in protective factors over time (enculturative values decreased in the border city, and optimism and social support/connection decreased in the interior city), though longitudinal trends in risk factors varied across sites. For example, in the border city we saw increases in acculturative stress and discrimination, while in the interior city there was no change in immigrant-related stress, but a small decrease in reported discrimination. We also identified high levels of anxiety, depression, and perceived stress reported by mothers at both study sites at the follow-up time period, and an increase over time in these measures in the border city, where they were assessed longitudinally. The symptoms of stress did not increase over time in the interior city, but were measured in a small sample at the follow-up time point. Finally, we found these mental health outcomes to be positively associated with acculturative stress and discrimination in the border city, and negatively associated with protective factors in both cities, though findings differed across time points.

## Discussion

We identified longitudinal changes in sociocultural stressors, protective factors, and mental health among Latina mothers across this period of a shifting sociopolitical climate following the candidacy announcement of President Trump. As hypothesized, our data support heightened levels of sociocultural stressors since President Trump’s political emergence, though changes were not always consistent across locations. Though we found consistent decreases in protective factors at both study sites, we found significant increases in reported stressors at the border but not in the interior city. Mental health also worsened at follow-up with significant increases in tiredness in the interior city, and increases in anxiety and perceived stress in the border city. Overall, we identified protective factors associated with reductions in mental health symptoms across both cities.

Differences between sites, particularly for changes in discrimination and stress over time, may stem from the use of different measures, but also from historical differences in policies and cultures between sites. For example, women in the interior city often reside or work in conservative neighborhoods where discrimination levels may have been higher over time. In fact, we reported high levels of discrimination in 2014 experienced by some of these same mothers [19]. The border city, conversely, has been a more traditional immigrant destination with a history of community support and integration [38], and discrimination may have only recently increased. For example, CA reported a 52% increase in hate crimes towards Latinos between 2016–2017 [4]. Interestingly, the decreased discrimination reported in the interior city is consistent with findings from a nationally representative study of White Americans, who self-reported a decline in prejudice towards Latinx people after Trump’s political emergence [15], potentially in rejection of rising racist presidential rhetoric [15]. Alternatively, Latina mothers in the interior city may simply have been more hesitant to report discrimination under the more hostile political climate.

Our most striking and consistent finding is a reduction in protective factors across both study sites. This finding is important for interpreting changes in family stress as a whole, as the family stress model posits that the presence of resources to cope with stress affects a family’s overall response to stress [10]. This reduction is particularly concerning, since factors like familism and social support are important for maintaining mental health, particularly in high stress environments [11, 39] and especially in Latinx communities [40]. Interestingly, most of these decreasing factors were related to the value of family, an important source of social support in Latinx families [40]. In particular, the scales of family referent and respect that decreased over time both focused on intergenerational relationships, especially relationships between children and parents, normally a strong part of Mexican culture associated with resilience [41]. While part of this loss may be simply due to time away from extended families, the observed decrease in protective factors, regardless of years in the US, may also relate to recent policies towards immigrants. Reduced family support could result from diminished social networks or loss of community integration as family and friends get deported [42]. Further, we note that years lived in the US was associated with perceived stress and anxiety at baseline but not follow-up, suggesting additional external factors (e.g. heightened racism) may influence mental health after Trump’s rise to presidency.

In addition to loss of protective factors, we report changes in discrimination, acculturative stress, and mental health over time. Changes in these factors, unlike the protective factors, may not be related necessarily to a “Trump Effect,” but perhaps increased awareness of discrimination and acculturative stress over time lived in the US. Additionally, changes in children’s developmental timing over the course of the study (6 months to 3 years) may have influenced maternal mental health. Previous work has identified the first year as the time when mothers are most susceptible to depressive episodes (approximately 10–19% prevalence) [43, 44]. Even though the rate drops after the second year, mothers continue to experience maternal depressive episodes at high rates until children reach approximately 10 years old, especially if they had previous episodes [45]. Thus, mothers may be experiencing different levels of depression symptoms across the first few years, however, depression symptoms normally decrease, and in the current study symptoms increased on average, suggesting this effect was not due to increase in children’s age alone. Regardless of why the changes occurred, the increased stress and decreased mental health over time may have harmful implications for all mothers, but may be especially dangerous for pregnant women, who are at risk for preterm birth outcomes. As other studies have shown [7, 46], preterm birth rates increased among Latina women in the period following the 2016 election in NY and nationwide, corresponding to the period of shifting policies and regulation of immigration in the new political regime.

To contextualize the levels of depression and anxiety in our study we compared levels across our studied cities to levels reported by mothers in the general population and Latinx groups in the past. The high proportion of borderline/clinically diagnostic levels of depression varied across our study sites (29% and 40% for depression in the interior and border cities respectively), but more similar levels of anxiety were found in both cities (39.5% and 34.0%, respectively) at follow-up. These levels are much higher than those reported in the general population, where incidence rates of maternal depression (after the first year of the child’s life) in Britain are usually as low as 7–8 per 100 person-years, as measured until the child is 12 years old, and the prevalence rate in the US is 10% [44, 47]. There are also racial/ethnic disparities in depression with Latina women reporting clinically higher levels of depressive symptoms than their White counterparts [48, 49]. In Mexican Americans in decades past, depressive symptoms and anxiety levels (e.g. Study of Latinos, SOL, 2007–2011) were lower than levels reported in our study, with 22.3% reporting high depression, and mean anxiety levels averaged 17 (out of a scale ranging 10–40) [50]. In more recent time periods, such as a Seattle-area study of largely undocumented Latina mothers (2018–2020) [51], levels were higher and very similar to our findings, with 29% reporting moderate to severe depressive symptoms and 32% reported moderate to severe anxiety symptoms. Thus, the rates reported in our study populations are high compared to the general population and Mexican Americans in the past, though similar to other studies investigating depressive and anxiety symptoms in mothers of Mexican descent in the current sociopolitical era.

Our regression analyses revealed consistent associations over time between acculturative stress and worse mental health in the border city, suggesting that acculturative stress is consistently harmful, regardless of years lived in the US, maternal age, or shifts in political climate. In contrast, discrimination was associated with greater perceived stress only at baseline, despite higher levels of discrimination reported at follow-up. Possibly, participants grew accustomed to discriminatory experiences with more time lived in the US, such that it had a weaker association with mental health, which is supported by Pew research showing older generations of Latino immigrants tend to report less discrimination than newer (first and second) generations [52]. Similarly, a protective effect of Mexican orientation on perceived stress was found only at baseline, implying that the benefits of a strong cultural orientation may have eroded over time. While our data cannot distinguish between a “Trump effect,” per se, versus more years lived in the US in contributing to this decrease, we note that the majority of our sample in the border city were long-term US residents of over 20 years on average, and only less than 3 years had lapsed between sample collection time points. Thus, we infer that reduced Mexican orientation in such a short period of time may be related to the dramatic sociopolitical shifts rather than simply elapsed time. On the other hand, Mexican orientation has shown to decrease over generations in the US [53], and over formative years of adolescence [54], but less research has addressed short-term shifts in adults, where cultural values appear to be relatively stable [9]. Regardless, a loss of this protective factor during this precarious period would likely be harmful for this population. In contrast, we saw a consistent effect over time for the cultural traditions of valuing intergenerational support and wisdom (respect and family reference), which were both protective and highlight more specifically the consistent role of familial support in the mental health of Latina mothers.

In the interior city, we found little evidence that our measured stressors were associated with perceived stress, anxiety, or depressive symptoms at follow-up, which may be related to small sample sizes at this time point. However, we saw strong and significant associations with protective factors, where social support/social connection and optimism were both associated with lower levels of perceived stress, suggesting these factors are important buffers for mental health, even if their presence decreased over time. In analyzing symptoms of stress over time, we found both immigrant-related stress and discrimination stress to associate with many reported symptoms of stress at baseline, but few at follow-up, where sample sizes were much smaller. However, we find it intriguing that discrimination is one of the only measures associated with anger/frustration and fears at follow-up, a time period when many mothers qualitatively expressed increasing fears as a result of Trump’s election and rising presence of MAGA hats, representing anti-immigrant sentiments.

Our study is strengthened by the inclusion of two parallel study sites–near the Mexican border and interior US–which allowed for comparison of similar sociocultural risk and protective factors across different geographic contexts. While our measures across study sites differed, we captured similar concepts of immigrant stress and protective factors in both sites. For example, in following family stress theory, we focus less on major life events than on daily hassles (e.g., discrimination stress, immigrant-related and acculturative stressors) which tend to have a strong association with psychological health outcomes [55]. Further, we controlled for important confounders of years in the US and maternal age effects in regressions. However, interpretation of our findings is limited by self-reported measures and small convenience samples, highlighting the need for follow-up studies in larger cohorts. Additionally, while our focus is on the mother, our intention is to ultimately understand how this stress impacts the entire family, the key unit of family stress theory [56], as a mother’s mental health is key to her family’s health (Fig 1).

## Conclusions

This study indicates the extent to which a period of shifting political leadership and policies may affect the mental health of a vulnerable population in the US. We find that racial/ethnic discrimination and acculturative stress have increased over time in a border city and are persistently harmful for Latina mental health. Given decreasing protective factors over time, targeted interventions to increase these factors and increase screenings could offset declines in mental health. This is especially important for Latina mothers, who have two to three times the risk of perinatal depression and anxiety relative to the general US population [9]. Continued research on best practices for reducing stressors and enhancing protective factors for minority health is warranted.

## Supporting information

S1 Table Summary of coefficient alphas and questions in sociocultural stressors, protective factors, and symptoms of stress measured in interior city. *α*^‡^ is Cronbach’s alpha for all items listed in each scale.
(DOCX)

S2 Table Logistic regression models of maternal sociocultural stressors and protective factors explaining symptoms of stress at baseline and follow-up in the interior city (*n*’s = 79–81 baseline, *n*’s = 38–39 follow-up). The models are adjusted for maternal age and years in the US. **Bolded** values are *p*<0.05. *Italicized* values are *p*<0.10. Symptoms of stress represent symptoms mothers reported in response to the question, “Which of the following symptoms do you experience usually as a result of stress?”.
(DOCX)
