# Supplementary material for: Changes in sociocultural stressors, protective factors, and mental health for US Latina mothers in a shifting political climate
Source: PLoS One. 2022 Aug 25;17(8):e0273548. doi: 10.1371/journal.pone.0273548 (PMC9409595; doi:10.1371/journal.pone.0273548)
Supplement: S1 Table — α‡ is Cronbach’s alpha for all items listed in each scale. (DOCX) [file pone.0273548.s001.docx]

Supplemental Table 1.0. Summary of coefficient alphas and questions in sociocultural stressors, protective factors, and symptoms of stress measured in interior city.

|  | **α^‡^**  **(95% CI)** | **# of Items** | **Questions (English Translations)** |
| --- | --- | --- | --- |
| **Maternal Reported Stress Scales** | | | |
| Immigrant-related stress | 0.64  (0.53, 0.76) | 10 | When I emigrated to the US I felt stressed because: (yes/no)   1. Couldn’t afford to bring family when emigrated 2. No legal documentation when emigrated 3. Separated from spouse or children when emigrated 4. Lost contact with my family when emigrating   During the past year I felt stressed because: (yes/no)   1. I could not communicate with others 2. My partner or a close family member was arrested. 3. My partner or a close family member was deported. 4. My family or I had difficulty adjusting to American customs. 5. My family live far away. 6. My partner does not have legal documents. |
| Discrimination stress | 0.69  (0.60, 0.78) | 7 | How often did you feel that you: (yes/no)   1. Were discriminated against at your job? 2. Were treated as if you were less than other Americans 3. Were discriminated against at the doctor’s office or hospital 4. Experienced discrimination in your neighborhood because you are an immigrant 5. Were treated unfairly because you are Latina? 6. Have seen friends treated badly because they are Latina? 7. People dislike you because you are Latina? |
| **Maternal Protective Factor Scales** | | | |
| Social Support and Connection | 0.59  (0.45, 0.72) | 11 | Please tell me whether you Strongly Agree, Agree, Disagree, Strongly Disagree:   1. I have had difficulty making friends in Nashville. 2. I have difficulty seeing my friends or family because I lack transportation. 3. I have found emotional support through my church. 4. I had family members near where I live but I had no relationship with them. 5. I have friends or family with whom I can talk about my feelings or problems. 6. I have friends or family who can help with financial troubles. 7. On average, how many times do you talk on the telephone with family, friends, or neighbors who live near you in the US? 8. On average, how many times do you talk on the telephone with family, friends, or neighbors who live in your country of origin? 9. On average, how often do you get together with friends or relatives? 10. On average, how often do you attend church or religious services? 11. On average, how often do you attend meetings of the clubs or organizations you belong to? |
| Life Orientation Test-revised (LOT-R, Optimism) | 0.43  (0.25, 0.61) | 6 | Please answer whether you agree a lot, a little, neither agree nor disagree, disagree a little, or disagree a lot with each question.  1.  In uncertain times, I usually expect the best.  [2.  It's easy for me to relax.] (filler) 3.  If something can go wrong for me, it will. (reverse) 4.  I'm always optimistic about my future. [5.  I enjoy my friends a lot.] (filler) [6.  It's important for me to keep busy.] (filler) 7.  I hardly ever expect things to go my way. (reverse)  [8.  I don't get upset too easily.] (filler) 9.  I rarely count on good things happening to me. (reverse)  10.  Overall, I expect more good things to happen to me than bad. |
| **Maternal Mental Health Measures** | |  |  |
| Hospital anxiety and depression scale (HADS) | 1. 0.64   (0.51, 0.75)  (D) 0.48  (0.29, 0.63) | 7  7 | Tick the box beside the reply that is closest to how you have been feeling in the past week.   1. (A) I feel tense or 'wound up' 2. (D) I still enjoy the things I used to enjoy (reverse) 3. (A) I get a sort of frightened feeling as if something awful is about to happen 4. (D) I can laugh and see the funny side of things (reverse) 5. (A) Worrying thoughts go through my mind 6. (D) I feel cheerful (reverse) 7. (A) I can sit at ease and feel relaxed (reverse) 8. (D) I feel as if I am slowed down 9. (A) I get a sort of frightened feeling like ‘butterflies’ in the stomach 10. (D) I have lost interest in my appearance 11. (A) I feel restless as I have to be on the move 12. (D) I look forward with enjoyment to things (reverse) 13. (A) I get sudden feelings of panic 14. (D) I can enjoy a good book or radio or TV program (reverse)   Note: (A) indicates part of Anxiety scale, (D) indicates part of depression scale. Reverse indicates reverse coding such that high values indicate higher anxiety or depression. |
| Perceived Stress Scale (PSS) | 0.38  (0.20, 0.57) | 10 | For each question choose from the following alternatives: 0-never, 1-almost never, 2-sometimes, 3-fairly often, 4-very often  l. In the last month, how often have you been upset because of something that happened unexpectedly?  2. In the last month, how often have you felt that you were unable to control the important things in your life?  3. In the last month, how often have you felt nervous and stressed?  4. In the last month, how often have you felt confident about your ability to handle your personal problems?  5. In the last month, how often have you felt that things were going your way?  6. In the last month, how often have you found that you could not cope with all the things that you had to do?  7. In the last month, how often have you been able to control irritations in your life?  8. In the last month, how often have you felt that you were on top of things?  9. In the last month, how often have you been angered because of things that happened that were outside of your control?  10. In the last month, how often have you felt difficulties were piling up so high that you could not overcome them? |
| Maternal Symptoms of Stress | -- | 1 | Which of the following symptoms do you experience usually as a result of stress? Indicate all of the symptoms you experience.  1) Tiredness  2) Sickness  3) Low energy  4) Frustration/anger  5) Depression/anxiety due to stress  6) Fear  7) Feeling as if they were aging too fast  8) Trouble falling asleep  9) Trouble staying asleep  10) None |

α^‡^ is Cronbach’s alpha for all items listed in each scale, using baseline data from the total dataset.
