# Supplementary material for: Changes in sociocultural stressors, protective factors, and mental health for US Latina mothers in a shifting political climate
Source: PLoS One. 2022 Aug 25;17(8):e0273548. doi: 10.1371/journal.pone.0273548 (PMC9409595; doi:10.1371/journal.pone.0273548)
Supplement: S2 Table — The models are adjusted for maternal age and years in the US. Bolded values are p<0.05. Italicized values are p<0.10. Symptoms of stress represent symptoms mothers reported in response to the question, “Which of the following symptoms do you experience usually as a result of stress?”. (DOCX) [file pone.0273548.s002.docx]

Supp Table 2: Logistic regression models of maternal sociocultural stressors and protective factors explaining symptoms of stress at baseline and follow-up in the interior city (*n*s=79-81 baseline, *n*s=38-39 follow-up).

|  |  | ***Symptoms of Stress*** | | | | | | | | | | | | | | | | | | | | | | | | | | | | | |
| --- | --- | --- | --- | --- | --- | --- | --- | --- | --- | --- | --- | --- | --- | --- | --- | --- | --- | --- | --- | --- | --- | --- | --- | --- | --- | --- | --- | --- | --- | --- | --- |
|  |  | **Tiredness** | | **Sickness** | | | **Aging too quickly** | | | **Energy Level** | | | | | **Staying Asleep** | | | | **Falling Asleep** | | **Fear** | | | **Anger/**  **Frustration** | | | **Depression/Anxiety** | | | |  |
| **Maternal variables** | | Adj *R*^2^ | $B$(SE) | | Adj *R*^2^ | $B$(SE) | | Adj *R*^2^ | $B$(SE) | | Adj *R*^2^ | $B$(SE) | | | | Adj *R*^2^ | $B$(SE) | Adj *R*^2^ | | $B$(SE) | | Adj *R*^2^ | $B$(SE) | | Adj *R*^2^ | $B$(SE) | | Adj *R*^2^ | $B$(SE) |  |  |
| **Sociocultural Stressors**  **Immigrant-related stress** | | | | |  |  | |  |  | |  | |  |  | | |  |  | |  | |  |  | |  |  | |  |  |  |  |
| Baseline | | 0.037 | 0.29  (0.24) | | **0.099** | **0.67 (0.23)** | | **0.068** | **0.53 (0.24)** | | 0.010 | | -0.10 (0.25) | **0.104** | | | **0.50 (0.24)** | 0.079 | | 0.17 (0.25) | | 0.010 | 0.18 (0.24) | | 0.064 | 0.30 (0.25) | | **0.076** | **0.49 (0.24)** |  |  |
| Follow-up | | 0.029 | 0.02  (0.33) | | 0.092 | 0.66 (0.47) | | 0.056 | 0.59 (0.48) | | 0.009 | | -0.11 (0.49) | *0.174* | | | *0.85 (0.46)* | 0.002 | | 0.04 (0.50) | | 0.031 | -0.13 (0.47) | | 0.062 | -0.05 (0.48) | | 0.034 | 0.12 (0.49) |  |  |
| **Discrimination stress** | | |  | |  |  | |  |  | |  | |  |  | | |  |  | |  | |  |  | |  |  | |  |  |  |  |
| Baseline | | 0.023 | 0.13  (0.22) | | **0.101** | **0.62 (0.21)** | | 0.019 | 0.21 (0.22) | | 0.014 | | 0.15 (0.23) | 0.075 | | | 0.29 (0.22) | *0.115* | | *0.42 (0.22)* | | 0.003 | 0.06 (0.22) | | **0.143** | **0.63 (0.21)** | | **0.096** | **0.53 (0.22)** |  |  |
| Follow-up | | *0.126* | *-0.42*  *(0.22)* | | 0.048 | 0.17 (0.36) | | 0.062 | 0.49 (0.36) | | 0.063 | | 0.51 (0.35) | 0.119 | | | -0.08 (0.35) | 0.006 | | 0.01 (0.37) | | *0.144* | *0.66 (0.33)* | | **0.220** | **0.78 (0.32)** | | 0.043 | 0.05 (0.36) |  |  |
| **Protective factors**  **Social support/connection** | | | | |  |  | |  |  | |  | |  |  | | |  |  | |  | |  |  | |  |  | |  |  |  |  |
| Baseline | | **0.069** | **-0.19**  **(0.09)** | | **0.070** | **-0.22 (0.09)** | | 0.017 | -0.08 (0.10) | | **0.063** | | **-0.21 (0.10)** | **0.109** | | | **-0.20 (0.09)** | **0.128** | | **-0.21 (0.09)** | | *0.038* | *-0.15 (0.09)* | | 0.091 | *-0.19 (0.09)* | | 0.057 | -0.15 (0.10) |  |  |
| Follow-up | | 0.044 | 0.08  (0.11) | | 0.052 | 0.10 (0.16) | | 0.016 | -0.02 (0.16) | | 0.008 | | 0.03 (0.16) | 0.157 | | | 0.25 (0.15) | 0.001 | | -0.01 (0.16) | | *0.129* | *-0.29 (0.15)* | | 0.069 | 0.08 (0.16) | | 0.033 | -0.02 (0.16) |  |  |
| **Optimism** | |  |  | |  |  | |  |  | |  | |  |  | | |  |  | |  | |  |  | |  |  | |  |  |  |  |
| Baseline | | 0.026 | -4.14e-03 (0.02) | | 0.002 | -0.01 (0.02) | | 0.010 | 1.65e-03 (0.02) | | 0.011 | | -4.63e-03 (0.02) | 0.080 | | | -0.03 (0.02) | 0.082 | | -0.01 (0.02) | | 0.007 | -0.01 (0.02) | | 0.049 | -0.01 (0.02) | | 0.059 | -0.03 (0.02) |  |  |
| Follow-up | | 0.074 | -0.03  (0.03) | | 0.059 | -0.03 (0.04) | | 0.029 | -0.03 (0.04) | | 0.009 | | -0.01 (0.04) | 0.105 | | | -0.03 (0.04) | 0.022 | | -0.03 (0.04) | | 0.040 | 0.02 (0.04) | | 0.064 | -0.01 (0.04) | | 0.046 | -0.03 (0.04) |  |  |

The models are adjusted for maternal age and years in the US. **Bolded** values are *p*<0.05. *Italicized* values are *p*<0.10. Symptoms of stress represent symptoms mothers reported in response to the question, “ Which of the following symptoms do you experience usually as a result of stress?”
